# Conjunctival Pediatric-Type Follicular Lymphoma in a Young Male: A Case Report and Literature Review

**DOI:** 10.7759/cureus.22023

**Published:** 2022-02-08

**Authors:** Abdullah F Alnaim, Abrar Alhawsawi, Abdulaziz AlSomali, Raneem Jannadi, Sana M Alsolami, Hammam A Alotaibi

**Affiliations:** 1 Ophthalmology, Dhahran Eye Specialist Hospital, Dhahran, SAU; 2 Ophthalmology, University of Jeddah, Jeddah, SAU; 3 Ophthalmology, King Faisal University, AL Hasa, SAU; 4 Anatomical Pathology, Dammam Regional Laboratory and Blood Bank, Dammam, SAU; 5 Research Center, Prince Sultan Military Medical City, Riyadh, SAU

**Keywords:** ocular cancer, conjunctival growth, head and neck cancer, orbital malignancy, pediatric-type follicular lymphoma

## Abstract

Pediatric-type follicular lymphoma is a disease that affects the lymph nodes of the head and neck in the adult and pediatric patient groups. Ocular involvement is exceedingly rare, especially in the pediatrics age group; therefore, keeping a high clinical suspicion is warranted.

Here, we report a rare conjunctival pediatric-type follicular lymphoma in a 15-year-old boy presenting with progressive swelling over the medial aspect of the left bulbar conjunctiva for two months. On examination, the mass was firm, mobile, well encapsulated, wide-based, and had a negative transillumination. An excisional biopsy was performed, and histopathological examination and immunohistochemistry studies revealed lymphoid tissue that was positive for CD20, CD79a, BCL6, and CD10; and negative for BCL2 and MUM1. The CD21 and CD23 positivity highlighted the presence of an expanded follicular dendritic cell meshwork. The patient was diagnosed with conjunctival pediatric-type follicular lymphoma and referred to an oncology center for further examination and treatment.

This lymphoma is rare, requiring high clinical suspicion, and thus, reporting the case detail is important and valuable for ophthalmologists and general pediatrics practitioners alike.

## Introduction

Lymphoma is one of the most prevalent adult orbital malignancies, representing 10% of all orbital tumors, and around 2% of all nodal and extra-nodal lymphomas [1,2]. Of all childhood systemic lymphomas, no more than 2% are pediatric-type follicular lymphoma [3]; this type is seen infrequently in young adults. Pediatric-type follicular lymphoma is classified as a novel variant of follicular lymphoma in the World Health Organization (WHO) classification of lymphomas. This disease predominantly affects males and is most commonly located in the head and neck region [3,4]. We describe a rare case of a well-circumscribed epibulbar conjunctival pediatric-type follicular lymphoma in a young male.

## Case presentation

This report was approved by the appropriate institutional review board (IRB), and the work was carried out in accordance with the code of ethics of the world medical association (Declaration of Helsinki).

**Figure 1:**
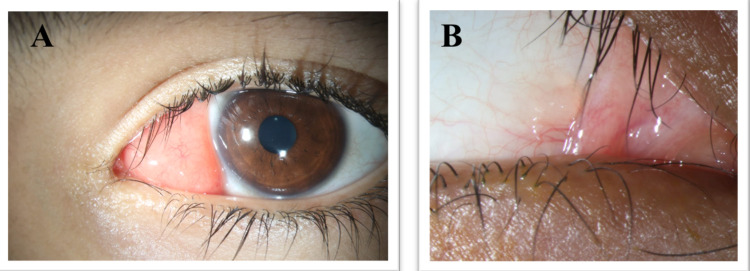
Conjunctival mass on the medial aspect of the left (A) and the right (B) eyes of the patient

A 15-year-old otherwise healthy boy presented to the emergency department in Dhahran Eye Specialist hospital with a two-month history of progressive swelling over the medial canthus of the left eye. On examination, a pink conjunctival mass over the medial aspect of the left eye was seen. The mass was firm, well encapsulated, mobile, wide-based, and had negative transillumination (Figure 1A). The right eye had a small, firm, and mobile conjunctival mass on its medial aspect (Figure 1B). Other ocular structures were unremarkable. No palpable lymph nodes were detected during the examination.

**Figure 2:**
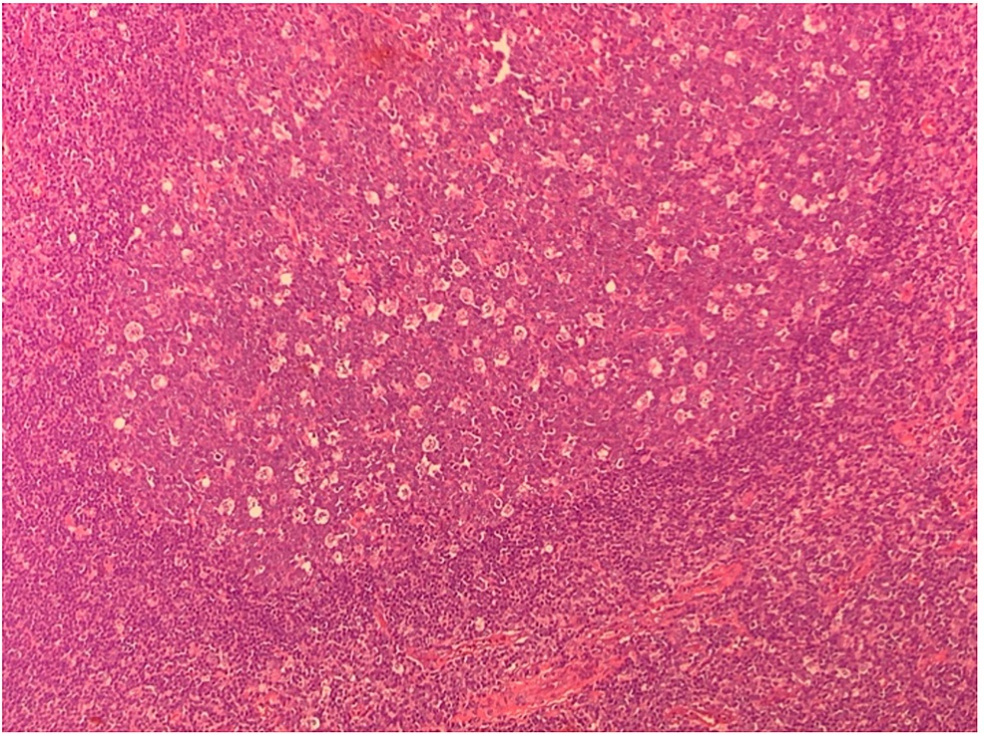
Histopathology of biopsy demonstrating a starry sky appearance

Anterior segment optical coherence tomography (OCT) was performed and revealed no retinal extension. An excisional biopsy of the lesion in the left eye was performed, and histopathological examination revealed lymphoid tissue with expanded and irregular serpiginous follicles, minimal mantle cell zone, and reduced interfollicular space. The germinal centers were predominantly composed of blastoid cells with numerous mitotic figures and multiple tingible body macrophages, giving the germinal centers a "starry sky" appearance. The blastoid cells were intermediate in size and had nuclear pleomorphism and multiple nucleoli. A few centrocytes and plasma cells were also present within the germinal centers. Reactive follicles were present at the periphery of the specimen (Figure 2).

**Figure 3:**
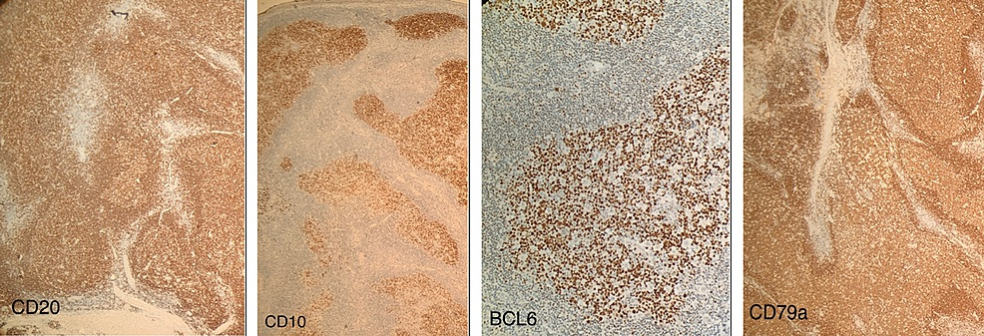
Immunohistochemistry of biopsy positive for CD20, CD79a, BCL6, and CD10 indicating lymphoma

**Figure 4:**
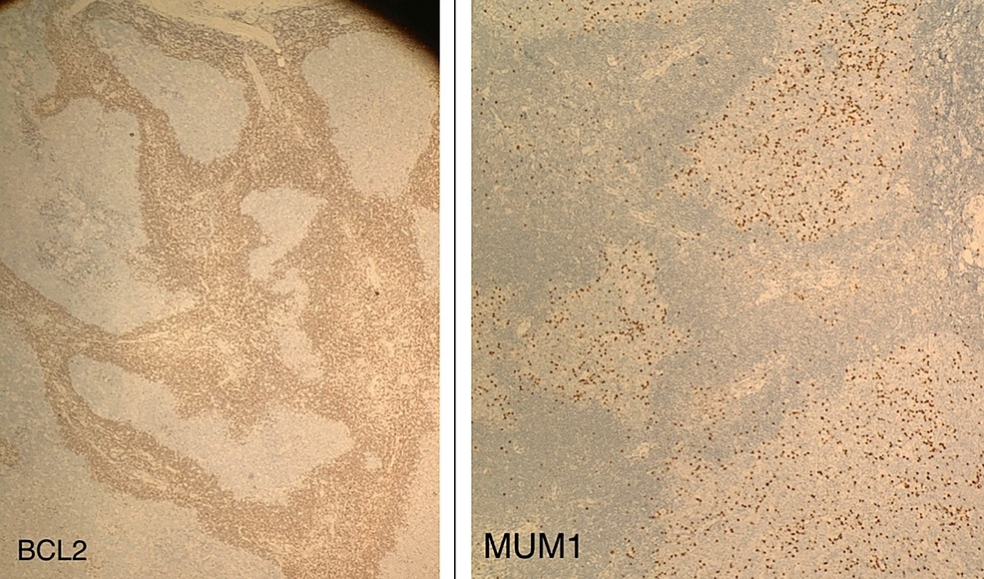
Immunohistochemistry of biopsy is negative for BCL2 and MUM1

Immunohistochemistry (IHC) examination was positive for CD20, CD79a, BCL6, and CD10 (Figure 3), and negative for BCL2 and MUM1 (Figure 4). The interfollicular spaces contained T-cells (CD3+). The CD21 and CD23 positivity highlighted the presence of an expanded follicular dendritic cell meshwork. The Ki-67 proliferation index within the germinal centers was approximately 95%. The patient was diagnosed with conjunctival pediatric-type follicular lymphoma and referred to the oncology center for further examination and treatment. Follow-up at one year post-surgery revealed no recurrence.

## Discussion

Conjunctival pediatric-type follicular lymphoma is a rare entity. Only seven cases have been reported in the literature summarized in Table 1 [5-11]. All cases reported were males with ages ranging from six to 21 years. Bilateral involvement was observed in three of the eight cases (two documented previously, the third is presented herein), and the medial bulbar conjunctiva was affected in seven of the eight cases. The duration from onset to presentation ranged from two to six months.

**Table 1:** Summary of the case reports of conjunctival follicular lymphoma in children and young men (n=11) cm^3^: cubic centimeter, IHC: immunohistochemistry, mm^2^: millimeter squared, N/A: not applicable

﻿Author(s)	﻿Gender/age	﻿Affected eye	﻿Gross description	﻿IHC markers	Ki-67	﻿Treatment and follow-up
﻿Gaffar et al. 2010 [5]	﻿Male/ 6 years	﻿Right	﻿Encapsulated tan-brown tissue measuring 6.0×3.0×2.5 mm^3^	﻿Positive: CD20, BCL6, and CD10; Negative: TdT, BCL2, and S-100	﻿>90%	﻿Excisional biopsy with no recurrence after three years
﻿Nazarullah et al. 2016 [6]	﻿Male/ 10 years	Bilateral	﻿Pink mobile nodular lesions (left: 6.2×4.3 mm^2^, right: 4.8×2.5 mm^2^)	﻿Positive: CD20, CD10, and BCL6; Negative: BCL2	﻿>90%	﻿Excisional biopsy. No documentation of follow-up duration
﻿Perry et al. 2012 [7]	﻿Male/ 21 years	Left	﻿N/A	﻿Positive: CD20, CD23, and CD10; Negative: BCL2	﻿>75%	﻿Excisional biopsy with no recurrence after eight months
﻿Rodriguez Torres et al. 2016 [8]	﻿Male/ 11 years	Left	﻿Tan gray mucosal soft tissue (0.8×0.6×0.3 cm^3^)	﻿Positive: CD20, CD10; Negative: BCL2	﻿>30%	﻿Excisional biopsy and radiotherapy (30 Gy) with no recurrence after two years
﻿Taghipour Zahir et al. 2013 [9]	﻿Male/ 12 years	Right	﻿Nodular lesion	﻿Positive: CD20, BCL2, and BCL6	NA	﻿Excisional biopsy with no recurrence after nine months
﻿Wall et al. 2015 [10]	﻿Male/ 10 years	Bilateral	﻿2 mm oval fleshy well-circumscribed lesion	﻿Positive: CD20, CD10, and BCL6; Negative: BCL2	﻿>90%	﻿Excisional biopsy followed by rituximab. No recurrence in the left eye and a smaller lesion in the right eye after 15 months
﻿AlSemari et al. 2020 [11]	﻿Male/ 18 years	Left	﻿Oval tan-smooth surface lesion (12×7.5 mm^2^)	﻿Positive: CD20, CD10, CD3, and BCL6; Negative: BCL2	﻿~75%	﻿Excisional biopsy with no recurrence after two years
Case presented herein	Male/ 15 years	Left	Well encapsulated with wide base, firm, mobile, and negative transillumination	Positive: CD20, CD79a, and BCL6; Negative: BCL2 and MUM1	﻿~95%	Excisional biopsy and referral to the oncology center for further examination and treatment

It is important to consider benign reactive lymphoid hyperplasia in the differential diagnosis. Microscopic examination of reactive lymphoid hyperplasia lesions reveals large interfollicular zones of various sizes and shapes, while in pediatric-type follicular lymphoma, the follicles are similar in configuration and arranged close to each other [11,12].

Histopathological evaluation and IHC studies are essential to diagnose lymphoma. IHC markers were positive for CD20 in all eight cases, for CD10 in seven of eight cases, and for BCL6 in six of eight cases; however, almost all the cases were negative for BCL2, which is predominantly seen in the adult population [5-11]. All patients underwent complete excision, and no recurrences were observed.

## Conclusions

A case of follicular lymphoma in a pediatric patient is documented in this report. Follicular lymphoma of the conjunctiva and sub conjunctiva in the pediatrics age group is rare and could be easily mistaken for a more common entity such as reactive lymphoid hyperplasia. High suspicion of such tumors in children is warranted to provide necessary early treatment. Failing to do so will have a detrimental effect on the morbidity and mortality of the child. Especially given the improved life expectancy when prompt treatment is provided, as evident by this case where treatment was provided at the time of diagnosis leading to preventing recurrence at one year. Immunohistochemistry has an important role to play in the differential diagnosis of the disease, and unique characteristics can be identified, thus aiding in the diagnosis and assessment in selecting the proper treatment regimen for the patient.
